# What influences where they seek care? Caregivers’ preferences for under-five child healthcare services in urban slums of Malawi: A discrete choice experiment

**DOI:** 10.1371/journal.pone.0189940

**Published:** 2018-01-19

**Authors:** Edgar Arnold Lungu, Amarech Guda Obse, Catherine Darker, Regien Biesma

**Affiliations:** 1 HIV/AIDS Section, UNICEF Malawi, Lilongwe, Malawi; 2 Health Economics Unit, School of Public Health and Family Medicine, University of Cape Town, Cape Town, South Africa; 3 Department of Public Health & Primary Care, Trinity College Dublin, Dublin, Ireland; 4 Department of Epidemiology and Public Health Medicine, Royal College of Surgeons in Ireland, Dublin, Ireland; UNICEF-Indonesia, INDONESIA

## Abstract

Access to and utilisation of quality healthcare promotes positive child health outcomes. However, to be optimally utilised, the healthcare system needs to be responsive to the expectations of the population it serves. Health systems in many sub-Saharan African countries, including Malawi, have historically focused on promoting access to health services by the rural poor. However, in the context of increasing urbanisation and consequent proliferation of urban slums, promoting health of children under five years of age in these settings is a public health imperative. We conducted a discrete choice experiment to determine the relative importance of health facility factors in seeking healthcare for childhood illnesses in urban slums of Malawi. Caregivers of children under five years of age were presented with choice cards that depicted two hypothetical health facilities using six health facility attributes: availability of medicines and supplies, thoroughness of physical examination of the child, attitude of health workers, cost, distance, and waiting time. Caregivers were asked to indicate the health facility they would prefer to use. A mixed logit model was used to estimate the relative importance of and willingness to pay (WTP) for health facility attributes. Attributes with greatest influence on choice were: availability of medicines and supplies (β = 0.842, *p<0*.*001*) and thorough examination of the child (β = 0.479, *p <0*.*001*) with WTP of MK3698.32 ($11) (95% CI: $8–$13) and MK2049.13 ($6) (95% CI: $3–$9) respectively. Respondents were willing to pay 1.8 and 2.4 times more for medicine availability over thorough examination and positive attitude of health workers respectively. Therefore, strengthening health service delivery system through investment in sustained availability of essential medicines and supplies, sufficient and competent health workforce with positive attitude and clinical discipline to undertake thorough examination, and reductions in waiting times have the potential to improve child healthcare utilization in the urban slums.

## Introduction

A significant body of evidence demonstrates that access to quality healthcare services promotes population health independent of other social determinants [1]. In particular, provision and subsequent utilisation of quality healthcare is integral to improving health outcomes, enhancing user satisfaction [2] and is pivotal in contributing to early childhood development [3]. Recent estimates suggest that there is potential to reduce under-five child mortality from the estimated 7.6 million in 2010 to 2.3 million by 2035 if countries accelerate coverage of child health interventions to their most optimal levels [4]. Most of these interventions, such as standard case management of childhood conditions, are delivered within the healthcare system. However, optimal benefits of cost-effective child health interventions are not realised particularly by the disadvantaged population groups due to limited access to and utilisation of healthcare services [5]. Ensuring adequate access to and utilisation of healthcare therefore remains central to child health, survival and development.

The current Malawi Health Sector Strategic Plan (HSSP) 2017–2022 and the National Child Health Strategy (2013–2018) indicate the need to promote utilisation of child health services [6,7]. While these and other child health strategy documents have identified the rural poor as a vulnerable population group [8], evidence from elsewhere [9–11] suggests the need to consider the urban poor residing in slums as an important group to target with child health interventions to improve child health outcomes. An urban slum, defined as an urban geographical entity lacking in one or more of the following five conditions: access to improved water, access to sanitation, durable housing, sufficient living area, and secure tenure [12] essentially presents risk factors for child morbidity and mortality. Evidence from studies in Kenya [9,13] and a multi-country study in India, Egypt, Kenya and Bangladesh [14] demonstrate that child mortality in the slums were much higher than the rural areas and national averages.

There is a paucity of epidemiological evidence on the burden of childhood conditions and utilisation of child health services in Malawi’s urban slums. However, with an annual urbanisation rate of 6.2%, Malawi is among the fastest urbanising countries in the world and in the context of urban poverty, up to 61% of residents in Malawi’s capital city were estimated to be residing in slum conditions [15]. A focus on urban health targeting urban slum residents is therefore imperative. A qualitative study in urban slums of Malawi among caregivers of children under five years indicate that health facility challenges such as negative attitudes of providers, lack of essential supplies, and suboptimal examination discouraged their subsequent use of child health services [16]. Granted that the healthcare system is an important determinant of health [1], understanding preferences of caregivers of children under five years of age in urban slums is integral to organising healthcare services to be responsive to their needs and priorities.

Evidence is sparse on caregivers’ relative valuations of health provider characteristics influencing seeking child healthcare services. Few studies have shown the relative valuations of healthcare system factors for various healthcare utilization decisions in Africa, such as maternal delivery care, sexual and reproductive health services for young people, and hospital quality [17–21].

This study aims to determine the relative importance of health facility factors influencing care-seeking decisions for under-five child healthcare services in urban slums of Malawi using a discrete choice experiment (DCE). DCEs are an important way to glean information about the priorities of users of services including healthcare. The use of DCEs in eliciting provider preferences for healthcare, thereby informing optimal organisation of health service, has been recognised and extensively used in high-income countries but its use in low- and middle-income countries is relatively low [22] albeit that it is recently growing. In Malawi, DCEs have been conducted in areas of human resources for health [23–24]; sexual and reproductive health for young people [25] and micro-health insurance [26]. To our knowledge, this is the first DCE focussing on preferences of child healthcare services in an urban slum population.

In Malawi, health services, including child health services, are predominantly provided by the public sector where they are free at the point of use. The public sector provides about 60% of healthcare services. Other providers are Christian Health Association of Malawi (CHAM–an umbrella body of health facilities owned and managed by Christian churches which operate on a not-for-profit basis hence charge user-fees for health services on a cost recovery basis); and the private health sector which charges user-fees on a profit basis [27].

## Materials and methods

We used a DCE to elicit caregivers’ stated preferences for child healthcare services. DCE is an attribute-based approach for eliciting preferences of consumers in hypothetical choice scenarios [5,28]. In a DCE, respondents are presented with a sequence of choice sets with two or more alternatives and asked to choose their most preferred alternative for each choice scenario. DCE is a rigorous technique for eliciting stated choices. It has increasingly been used to explore preferences of clients on various issues such as healthcare delivery, priority setting, and outcome measures [5,20,28]. Development of this DCE followed the five recognised steps of undertaking a DCE [29].

### Setting

This DCE was embedded in longitudinal cohort study that was conducted in three urban slums of Senti, Mgona and Ntandire in Lilongwe, the capital city and one of the fastest urbanising of Malawian cities. The longitudinal study entailed collecting information on: demographic and socioeconomic factors for individuals and households (only at baseline); childhood morbidity focussing on three common childhood conditions/symptoms in Malawi namely Acute Respiratory Infection (ARI), fever and diarrhoea; and healthcare seeking from a biomedical health provider. Data were collected over three time points (baseline, first follow up and second follow up) that were three to four months apart. This DCE was conducted immediately after the second (and final) follow up of the longitudinal study.

### Attributes and levels

Health facility related attributes that were important for caregivers were initially identified through a review of existing evidence from sub-Saharan Africa [1,14]. A qualitative study which was also part of the larger longitudinal study, was conducted prior to this DCE study to further identify and refine contextual attributes [16]. The qualitative study included 8 focus group discussions (FGDs) with caregivers of children under five years of age and 11 in-depth interviews (IDIs) with key informants (health workers, policy makers). Both FGDs and IDIs sought to explore healthcare system factors influencing utilisation of under-five child healthcare services. Factors that emerged as recurrent themes using content analysis were selected to constitute attributes for this DCE. Six attributes that describe a healthcare provider were identified to be the most important factors in determining child healthcare seeking, namely: distance to health facility; thoroughness of physical examination of the child; availability of medicines and supplies; attitude of health workers; waiting time; and cost of healthcare. Specific details of the qualitative study that informed attributes for this DCE are described elsewhere [16]

The levels of the attributes had to be plausible, and actionable while narrow enough to make competitive choices [29]. As with attribute identification, assigning attribute levels was informed by the qualitative inquiry. In addition, the longitudinal study informed levels for quantitative attributes of cost, waiting time, and distance to a health facility. Four of the six attributes in this DCE had three levels and two had two levels (Table 1).

**Table 1:** Attributes, levels and attribute description.

Attribute	Attribute level	Codes	Description
Distance to health facility	Half an hour	30	Distance as measured by the time it takes to walk to the facility in minutes
	1 hour	60	
	1 and half hours	90	
Availability of medicines and supplies	Medicines and medical equipment not available	0	Entails whether appropriate medicines and supplies for treatment of the child are available or not
	Medicines and medical equipment always available	1	
Waiting time	2 hours	2	Relates to waiting from time of arrival at health facility to completion of all treatment services required and going home
	3 and half hours	3.5	
	5 hours	5	
Attitude of health worker	Health workers do not treat with respect, do not listen carefully when client is explaining	0	Entails whether attitude of health worker attending to care giver is friendly and respectful or is rude
	Health workers are friendly and treat with respect, listen carefully when client is explaining.	1	
Thoroughness of physical examination of the child	Child not examined, health worker just writes as I explain and prescribes medication as I finish	0	Refers to how the child is examined and physically assessed by the health worker
	Superficial examination	1	
	Child examined thoroughly	2	
*Cost of healthcare	Free	0	Relates to costs of healthcare at the facility
	MK 600	600	
	MK1700	1700	

*At the time of conducting this DCE the prevailing exchange rate was 300 Malawi Kwacha to 1 United States Dollar

### Choice sets and questionnaire

Choice sets that were presented to respondents were generated using experimental design methods. Given 4 attributes with 3 levels and 2 attributes each with 2 levels, a full factorial design which consists of all combination of the attributes and levels, resulted in 324 possible scenarios (i.e. 3^4^ x 2^2^). These combinations were too many for respondents to make choices by comparing alternatives in each scenario [28]. Therefore, a D-efficient design, which reduced the number of scenarios to manageable and efficient choice sets, was generated using SAS statistical software package [30]. The final choice sets included nine pairwise combinations assuming main effects only. Typically, respondents can manage up to 16 choice set comparisons before fatigue sets in [29, 31].

**Fig 1:**
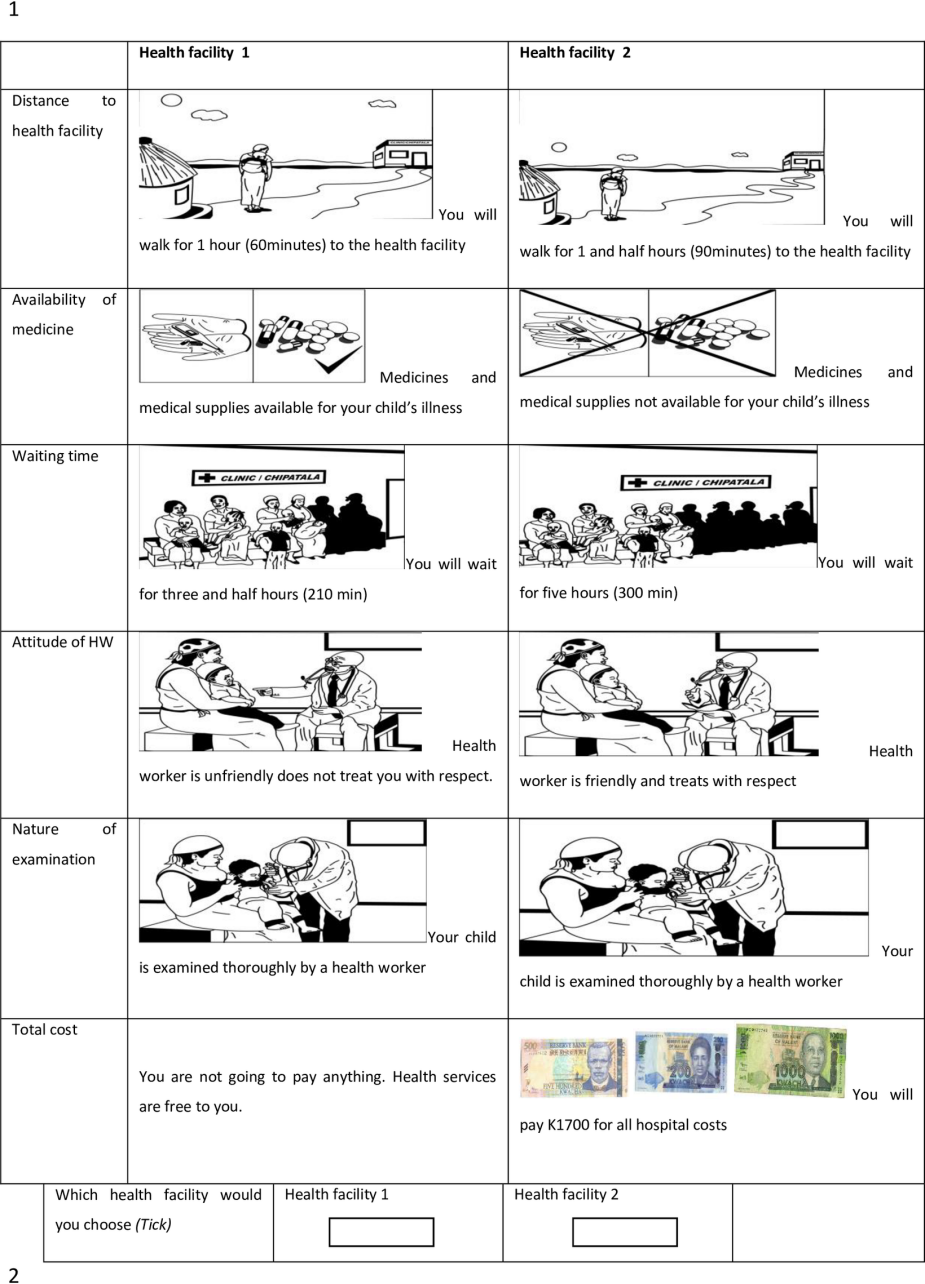
Example of DCE choice set pictogram presented to caregivers of children below five years of age.

An unlabelled choice format with two health facility choice alternatives (“health facility A” or “health facility B”) was used to arrange the choice sets in a questionnaire (S1 Text. DCE Questionnaire). An opt-out option was not included. Caregivers were requested to imagine that their child had an illness for which healthcare must be sought. In order to test the predictive power of the model, two holdout choice sets (i.e. additional choice questions made up by researchers and not generated by the experimental design procedures) were added to the nine leading to a total of 11 choice sets. The questionnaire also included socio-demographic characteristics such as: household monthly income; caregiver’s residence, age, occupation, and education level. To aid comprehension of choice sets among participants who were largely of low education levels, we used pictorial illustrations of choice sets (see Fig 1) designed by a local artist.

### Pre-testing of DCE questionnaire

Pre-testing of the questionnaire was undertaken in Mgona (one of the study sites) involving 30 caregivers who were part of the longitudinal study. The 30 participants were not eligible for the main DCE study. After conducting the choice tasks, questions were asked to get a better understanding of the complexity of the choice task for participants and to ascertain the usefulness of pictograms. The majority of participants (95%) involved in the pre-testing exercise indicated that the choice tasks were manageable and almost all (98%) indicated that the use of pictograms aided comprehension of the alternatives in the choice tasks.

Results from the pre-test exercise showed that signs of parameter estimates for the attributes were in the expected direction thus indicated preliminary confidence in respondent efficiency. Following the pre-test exercise, the following key modifications were made to improve on the logical flow of explanations and provide more clarification on some attributes: generating a full set of pictograms explaining every attribute level prior to presenting choice tasks; and explaining to participants that inclusion of cost attribute does not entail plans to introduce user fees in health facilities.

### Sampling and data collection

The longitudinal study used a sampling procedure that involved the following key steps: (i) conducting a household census in the three slum areas to enumerate all eligible households (with a child aged below five years, resident in an area for more than six months, with no plans to migrate out of study setting, and consent to participate in the study if selected) specifically collecting names of caregiver-child pairs from the household, and geographical description for purposes of identifying eligible households; (ii) allocating numerical codes to caregiver-child pairs and randomly selecting 543 caregivers using randomly generated numbers from Microsoft Excel with each area contributing to the sample according to its proportional representation to the total eligible numbers in all three study areas.

This DCE study involved a total of 340 participants who were randomly selected from 448 participants available during the second follow up of the longitudinal cohort study. Recruitment for DCE study participants was undertaken in February 2013. Random selection process entailed generating 350 random numbers from Microsoft excel (intentionally generating more random numbers than required for the sample size to cater for attrition) and matching them with the numeric codes (unique IDs) for caregivers in the longitudinal study database. A Pearsons Chi-square test was conducted to check if there were systematic differences in caregiver characteristics between the original sample and the one selected for this DCE, which could have compromised representativeness of the DCE sample to its population. The results indicated that there were no significant differences in caregiver characteristics.

Using the paper based DCE questionnaire, data were collected by trained enumerators using face-to-face interviewers in the participant’s home after obtaining informed consent following a process described subsequently. Each choice task was presented on a single sheet of paper. A caregiver was asked to imagine that her child was sick and given a choice of seeking child healthcare services from the two alternatives (“Health Facility A” or “Health Facility B”) described by the attributes, which of the two facilities would she choose if s/he must.

### Statistical analysis

Collected data were entered in SPSS 17, cleaned, and exported to Stata version 13 for analysis. Descriptive summary statistics were calculated for socio-demographic characteristics of the respondents. Choice data were estimated using mixed multinomial logit (MMNL) model. The MMNL model is chosen over the standard MNL since MNL model is constrained by the assumption of ‘independence from irrelevant alternatives’ (IIA). This assumption implies that there is no unobserved heterogeneity in preference and that utilities are not correlated across alternatives. On the contrary, parameter estimates are allowed to vary in MMNL to capture variations in taste and unobserved heterogeneity in alternative and choice sets [31–33].

Discrete choice models are based on random utility theory; in which, individuals are assumed to choose an alternative that gives them the highest utility or benefit from a list of alternatives [34, 35]. The utility individual *n* gets from alternative *i* is decomposable into deterministic (*βX_in_*) and error part [32]: as depicted below;

$$U_{in}={\beta X}_{in}+\varepsilon_{n}$$ (1)

The deterministic part of the utility is observable to the researcher and can be specified by attributes of the alternatives while the error term represents all other factors that influence utility of the respondent. Since, the researcher cannot get the information about the true utilities of the respondents, s/he can only observe probabilities of choice [36]. Therefore, a probabilistic utility function is specified based on an assumption of the error term. Faced with a binary health facility choice (Hospital A or Hospital B), the probability an individual chooses one hospital over another can be represented as:

$$Prob\;({Hosp}_{A}=1|x_{A},\beta )=Prob\;(U_{HospA}>U_{HospB});$$ (2)

which entails that Hospital A is chosen if the utility the individual derives from it is greater than the utility the individual gets from Hospital B.

We estimated two MMNL models. The first model consists of health facility attributes as sole explanatory variables while the second model includes interaction of the attributes with slum area. The second model is estimated to explore how slum area influenced valuation of health facility attributes. We assumed a normal distribution since the population preference distribution was unknown. The outcome variable (choice of health provider) was binary. Cost, distance, and waiting time were estimated linearly while the remaining variables were effects coded. The model was estimated based on 3000 Halton draws in STATA 13 [32,37]. The holdout choice sets were not included in the MMNL model but analysed separately.

Marginal willingness to pay (WTP), which represents caregivers’ mean monetary valuation of a unit change in health facility attribute levels is also estimated. This indicates how much the caregivers were willing to pay for the attribute level under consideration as compared to the reference level. [38,39]. The standard errors of the WTPs were estimated using Delta method.

### Ethical review

Ethical approval was granted by the National Health Sciences Research and Ethics Committee and the Trinity College Dublin’s Health Policy Management and Centre for Global Health Research and Ethics Committee under approval numbers NHSRC 895 and 27201107. Additionally, information about the study was explained to participants, and if they agreed to participate in the study they provided informed consent by either signing the consent form or finger printing (smearing finger with ink and pressing on the consent form) in the case of illiterate participants, prior to administering the DCE questionnaire.

## Results

### Characteristics of respondents

Of the 340 caregivers who were requested to participate in this DCE, 338 consented representing a 99% response rate. However, the final sample size was 301 after excluding incomplete questionnaires, hence a questionnaire completion rate of 89%. Table 2 presents study participants’ characteristics. About two in five respondents were from Ntandire. The mean age of caregivers was 29. Most of the respondents, 279 (92.7%), were married and had, on average, three children. Two hundred and eleven (70.1%) of the participants either had no education or primary school education. A total of 146 (48.5%) were unemployed.

**Table 2:** Characteristics of the DCE study participants from urban slums.

Variable	Frequency (%)
**Slum name**	
Senti	74 (24.6)
Mgona	103 (34.2)
Ntandire	124 (41.2)
**Caregivers age (Years)**	
16–25	103 (34.2)
26–30	72 (23.9)
31 +	127 (42.2)
**Monthly income**	
14000MK or less	94 (31.2)
15000-20000MK	113 (37.5)
More than 20000MK	95 (31.6)
**Education**	
No education / Primary	211 (70.1)
Secondary +	88 (29.5)
**Occupation**	
No activity	146 (48.5)
Self-employed / Small scale business	122 (40.5)
Employed	36 (12.0)
**Marital Status**	
Single/Widow/Divorced	22 (7.3)
Married	279 (92.7)

### Relative importance of health facility attributes

Table 3 presents the mixed logit regression results with healthcare facility attributes being the sole explanatory variables. The mean coefficients show, other things constant, the relative likelihood of choosing a health facility given the attribute level combinations. The magnitude of the coefficients indicates the relative importance of the attribute in the choice of health facility. Except for distance to health facility (time it takes to walk to health facility) and superficial examination, all other attributes were statistically significant.

**Table 3:** Mixed logit estimation result with and without interaction of a discrete choice experiment assessing healthcare facility attributes influencing utilization of under-five child healthcare services in urban slums of Malawi.

Attribute	***β*** SD	SE		***β*** SD	SE	
Cost	-0.001	0.000	***	-0.001	0.000	***
	-0.000	0.000	*	-0.000	0.000	*
Distance	0.001	0.001		0.003	0.004	
	0.008	0.001	*	0.009	0.004	**
Waiting time	-0.340	0.055	***	-0.413	0.118	***
	0.157	0.072	**	0.173	0.068	**
Medicine and equipment available (*reference—medicine and equipment unavailable*)	0.842	0.063	***	0.972	0.100	***
	0.357	0.058	***	0.367	0.063	***
Good attitude of health workers (*reference—health workers do not have good attitude*)	0.340	0.046	***	0.526	0.083	***
	0.304	0.068	***	0.291	0.072	***
Superficial examination (*reference—no examination*)	-0.024	0.057		-0.060	0.119	
	0.297	0.099	***	0.292	0.109	**
Thorough examination (*reference—no examination*)	0.479	0.099	***	-0.258	0.187	
	-0.039	0.15		0.105	0.206	
**Mgona interaction terms**						
Cost				0.000	0.000	**
Distance				-0.002	0.005	
Waiting time				0.154	0.144	
Medicine and equipment available (*reference—medicine and equipment unavailable*)				-0.112	0.108	
Good attitude of health workers (*reference—health workers do not have good attitude*)				-0.394	0.105	***
Superficial examination (*reference—no examination*)				-0.282	0.147	*
Thorough examination (*reference—no examination*)				0.577	0.236	**
**Ntandire interaction terms**						
Cost				0.000	0.000	
Distance				-0.002	0.005	
Waiting time				0.042	0.140	
Medicine and equipment available (*reference—medicine and equipment unavailable*)				-0.174	0.100	*
Good attitude of health workers (*reference—health workers do not have good attitude*)				-0.110	0.103	
Superficial examination (*reference—no examination*)				-0.001	0.144	
Thorough examination (*reference—no examination*)				0.132	0.244	
**Number of observations**	**5362**				**5362**	
**Log-likelihood**	**-1207.60**				**-1193.46**	
**Log-likelihood ratio *χ*^2^**	**214.67**				**213.63**	

β indicates the mean relative utility of an attribute relative to other attributes, SD is the standard deviation, and SE represents the standard error

***, **, * significant at 99%, 95%, and 90% confidence intervals respectively.

The estimated coefficients for all statistically significant attributes had the expected sign rendering theoretical validity of the direction of the effect. Caregivers’ strongly preferred health facilities that had medicines and equipment available and where their sick children were thoroughly examined Respondents valued a decrease in w in aiting time (β and good attitude of health workers equally (with opposite direction of influence) as the third most important attributes affecting their choice of a health facility. Cost of healthcare had the lowest effect on choice of health facility. The standard deviations of the mean coefficients indicate that there is preference heterogeneity among the caregivers.

An interaction model between attributes and slum area was undertaken with Senti as the reference slum area. The findings show that the interaction between Mgona and thorough examination was positive while it was negative for ‘good attitude of health workers’ and ‘superficial examination’. These values suggest that thorough examination was important to those from Mgona while superficial examination and good attitude of health workers were important to those from Senti. The interaction with Ntandire indicates that ‘medicine availability’ was less valued by those from Ntandire compared to those from Senti.

### Willingness to pay

Other things equal, respondents were willing to pay MK3698.32 ($11) (95% CI: $8–$13) for health facilities that had medicines and equipment available compared to the ones without medicines and equipment (see Table 4). Similarly, caregivers were willing to pay MK2049.13 ($6) (95% CI: $3–$9) and MK944.54 ($3) (95% CI: $1–$5) for thorough examination and superficial examination respectively over no examination of their child. This suggests that caregivers were prepared to pay MK1104.59 ($3) for a move from a superficial to a thorough examination of their child. Furthermore, caregivers were willing to pay MK1492.71 ($4.3) (95% CI: $3–$5) for an hour’s reduction in waiting time at the health facility.

**Table 4:** Willingness to pay for attributes of health facilities.

Attributes	Mean WTP	SE	95% CI
Distance	1.35	3.27	-5.05, 7.76
Waiting time	-1492.71	206.61	-1897.66, -1087.76
Medicine and equipment available	3698.32	488.08	2741.70, 4654.95
Good attitude of health workers	1495.06	202.10	1098.95, 1891.18
Type of examination			
Superficial examination	944.54	348.32	261.85, 1627.23
Thorough examination	2049.13	586.08	900.43, 3197.84

## Discussion

This DCE study assessed caregivers’ preferences for under-five child healthcare services in urban slums of Malawi. We found that caregivers in urban slums of Malawi mostly valued health facility attributes associated with clinical quality of healthcare such as availability of medicines and clinicians giving a thorough physical examination of their sick children. Non-clinical attributes including reduced in waiting time, positive attitudes of health workers, and reduced cost (including no cost), while ranking lower than clinical aspects, were still important to users of the health service.

The World Health Organisation recognises that responsiveness to people’s expectations is an essential intermediary goal of a health system and poor responsiveness can negatively affect utilization of services and the effectiveness of interventions [40]. Arguably a well-functioning health system delivers quality health services that attain patient/caregiver satisfaction and ultimately elicits demand for child healthcare services. Evidence from this study points to the need to strengthen child healthcare systems and in particular to prioritise efforts in promoting access to pharmaceutical supplies, adequate clinical examination of the child, along with improving health worker attitudes towards caregivers and reducing time in waiting for child healthcare services. Such considerations are crucial in child health programming such as the Integrated Management of Childhood Illnesses (IMCI) which relies heavily on these preferences for effective service utilization

The dearth of evidence from DCE studies in health, including child healthcare services in low-income country contexts has been acknowledged [28] and although application of DCEs has recently increased, those in child health remain few. This limits comparison of findings from this DCE with those from similar settings and with similar focus. This notwithstanding, our findings regarding important healthcare attributes are largely consistent with DCE studies undertaken in developing countries although relative significance of attributes sometimes differed with some studies. For example, a DCE study to elicit people’s preferences for attributes of public health facilities in South Africa also found availability of medicines to have the greatest marginal effect with other clinical attributes such as thorough examination and provision of expert advice being more valued than non-clinical quality care attributes including waiting time, staff attitude and treatment by doctors or nurses [18]. Similarly, a study in rural Ethiopia on women’s preference for obstetric care found availability of drugs and equipment as the most important attributes of a health facility, followed by provider type and attitude of health provider [41]. In Zambia, thorough examination was the most important health facility characteristic, followed by avoiding rude staff and availability of drugs [17]. In Malawi [42] and Tanzania [43], provider attitude and reliable access to adequate quantities of drugs and equipment were the two most important utility features of a health facility for reproductive health services for young people and obstetric services for women, respectively.

In contrast to our findings, other studies found that non-clinical attributes were regarded as more important than clinical attributes of healthcare. For example, a DCE study in urban Zambia found that avoiding a rude staff member to be more important than drug availability [17] and attitude of health provider had the highest valuations among attributes that included availability of drugs in two DCE studies among rural women in Tanzania [20,43].

Both waiting time and cost of healthcare negatively affected the choice of a health facility as indicated by negative coefficients in this study. Similar findings were noted in a DCE eliciting preferences for adult and child in-patient services in Zambia [17]. Furthermore, a review of DCE studies conducted in Europe to elicit preferences for health service attributes found waiting time to be one of the most important attributes [22]. Reducing waiting times has been integral to discourse in developed countries’ healthcare systems while it has arguably remained in the periphery in similar discourses in developing countries. Healthcare systems in developing countries are faced with numerous challenges to the extent that reduction in waiting times may not be of immediate primary importance, at least from the perspective of health workers, health planners and policy makers. However, our study reveals that even in a deprived setting, reducing waiting times in health facilities remains important to users hence warranting consideration in health planning.

Our study did not find evidence that distance was an important consideration in choice of health facilities. This contrasts with other DCE studies, which found distance as an important attribute albeit of a relatively lower weight in the choice of a health provider [41]. Moreover, distance to health facility, whether using Euclidean measures or travel time has conventionally been regarded as a crucial determinant of healthcare utilisation, and ultimately choice of a health provider [1]. Rationalising the relatively low importance of travel time in their DCE study in rural Ethiopia, Kruk *et al*. posited that where quality of healthcare is concerned people may take little consideration of the distance they have to travel [41]. A similar decision making mechanism may have been at play in responding to DCE questions in the context of our study in an urban slum setting, where distance may not have been considered as important in the presence of other health facility attributes, particularly those concerning clinical quality of care. Indeed, there is evidence that caregivers bypass their primary health facilities in favour of a similar level or higher level health facilities that are farther away but for which caregivers perceive that they offer health services of better quality [44]. Moreover, the Knowledge Network on Urban Settings (KNUS) for the WHO Commission on Social Determinants of Health posited that cost of healthcare and not distance may be the key determinant of health in urban settings [45].

An interactions model of geographical (slum) area revealed that relative to Senti, participants from Mgona valued thorough examination more whilst their valuations were relatively lower for superficial examination and good attitude of health workers. On the other hand, Ntandire participants, indicated less value for ‘medicine availability’ in comparison with those from Senti. It is probable that differential experiences of caregivers from different slums with their respective health facilities influenced their valuations for attributes where heterogeneity has been noted. To illustrate, it is probable that caregivers being served by a health facility that consistently has medicines and supplies available will value medicine availability attribute lesser than their counterparts served by a health facility experiencing stock outs of medicines. Indeed, others have argued that people draw upon their experiences of local health services or that of their families or friends when undertaking DCE exercises [41].

We found that caregivers in urban slums were willing to pay as high as MK3698 ($11) for a health facility with medicines and supplies available compared to the one without them. This valuation is about twice as high compared to thorough examination of the child (WTP = $6) and about three times that of attitude of health worker (WTP = $4). The level of private health facilities which study participants use would have user-fee charges at an average of MK1200 ($3.2) for treatment of a child with Acute Respiratory Infection symptoms. The WTP estimates in this study may thus be slightly overestimated for some attributes such as availability of medicines. Indeed, some arguments suggest that DCEs may overestimate WTP [5]. Therefore, as other authors argue, these WTP estimates should be interpreted with caution and are better viewed as valuations of relative importance of attributes included in the DCE study than as absolute measures [41].

It is argued that in contexts where health services are free at the point of use (as is the case in Malawi), the inclusion of cost attribute in DCEs may result in respondents providing protest responses under the impression that the intention of the study is to introduce user-fees in health facilities [46] which undermine the validity of the study findings. To counteract this, constant explanations and clarifications were provided intimating that the inclusion of cost was not intended to introduce user-fees, rather to understand monetary valuations of the health facility characteristics in the hypothetical choice scenarios.

Our study findings need to be interpreted in light of some limitations. We used a forced choice format for which there is a criticism that respondents are compelled to make choices even when none of the options in the choice set is desirable. More importantly in healthcare, caregivers may choose not to seek care at all. Thus, it is argued that forced choice formats overestimate parameter estimates. In addition, we estimated utility weights for health facility attributes only included in this study. While we are confident that an extensive review of literature and qualitative inquiry that we undertook effectively informed attribute selection and level definition, it is possible that we may have overlooked some health facility attributes.

The use of a rigorous methodological basis in an understudied population while capturing the currency of the urbanisation issue in Malawi; utilising pictograms to aid comprehension of choice sets; a very high response rate; and conducting a prior qualitative inquiry and extensive literature review for attribute selection and level definition, represent strengths for our study.

In conclusion, this study contributes evidence of the importance of health system-related factors in influencing care-seeking decisions for under-five child health services. The results of the study strongly suggest that availability of medicines and thoroughness of child examination are crucial in under-five child health service utilisation for caregivers in urban slums. Non-clinical features of the healthcare system including fostering positive attitude of health workers, reducing waiting times and costs should also be considered to improve user satisfaction. The Malawi Ministry of Health and its partners should therefore invest in strengthening child healthcare systems in urban slums and prioritise factors such as access to pharmaceuticals, clinical examination and professionalism of health workers. This will be critical in responding to the needs of under-five child healthcare service, potentially increase utilisation of under-five healthcare services and ultimately contribute to reduction in child morbidity and mortality in a rapidly growing urban slum population in Malawi.

## Supporting information

S1 Text DCE questionnaire. (DOCX)

S1 Dataset DCE dataset. (DTA)
