# Supplementary material for: What influences where they seek care? Caregivers’ preferences for under-five child healthcare services in urban slums of Malawi: A discrete choice experiment
Source: PLoS One. 2018 Jan 19;13(1):e0189940. doi: 10.1371/journal.pone.0189940 (PMC5774690; doi:10.1371/journal.pone.0189940)
Supplement: S1 Text — (DOCX) [file pone.0189940.s001.docx]

**DCE QUESTIONNAIRE**

**DCEQ CODE___________**

**Data collection tool for a DCE: relative significance of health system factors in influencing care seeking decisions for under-five child health services**

Slum Name : …………………………………………………………………………………………..

Date of Interview : …………………………………………………………………………………………..

Enumerator : …………………………………………………………………………………………..

Traditional Authority : …………………………………………………………………………………………..

**Introduction (*Read this and additional content on the Participant Information Leaflet***)

We would appreciate if you could answer some questions from this questionnaire. The questions pertain to significant factors for your choice of health care for your children under the age of five years. This survey is being carried out in three areas of Lilongwe city. There are no direct benefits for your participation in this research. However, the information you provide is important to inform stakeholders dealing in child health to improve delivery of child health services and promote the health of children especially in urban slum settings.

Participation is entirely voluntary. We wish to assure you of the confidentiality of the responses that you provide. The choices you make on this questionnaire will not affect you in any way.

Thank you for your participation

**Section A: Choice sets**

There are 11 choice sets in the subsequent sections. The choice sets relate to different types of a health facility from which you may seek care. Each choice set has two alternatives. Please, state/tick your preferred option for each set of choices.

Each alternative in the choices is constitutes of factors that describe the nature of a health facility. There are six factors in total (*Explain the six attributes and their levels using pictograms)*:

**Distance to health facility** – This entails how long it takes for you to reach the health facility. The distance is expressed in minutes (hrs) it would take to walk and it has three levels namely walking for 30, 60 and 90 minutes

**Cost to you** – describes the cost of accessing health services for your under-five child. There are three levels of this attribute namely; no cost to you, a cost of K600 and K1700.00.

**Availability of medicines and supplies** – relates to whether the medicines and supplies required to provide health care to your child are present at the facility or not.

**Waiting time** – this describes the time it would take for you from the point that you start waiting to be assisted to the time you have navigated through the health facility and received all the available help and ready to go back home. The waiting time also has three levels namely, waiting for 2 hrs, 3.5 hrs and 5 hrs.

**Attitude of health workers**: describes how health workers relate with you. Whether they are friendly and treat you with respect or they are unfriendly and seem not to care too much about you and your sick child.

**Nature of examination:** this entails how health workers examine your sick child. They may not examine the child or may examine superficially or do a thorough examination.

An example is provided below showing the choice made (*show the pictogram example)*.

**Example**

|  | **Health facility 1** | **Health facility 2** |
| --- | --- | --- |
| Distance to health facility | One hour | One and half hours |
| Availability of medicine | Medicine and equipment available | Medicine and equipment not available |
| Waiting time | 2 hours | 5 hours |
| Attitude of HW | Treat without respect | Treat with respect |
| Nature of examination | Superficial examination | Through examination |
| Cost to you | K1700 | Free |

| Which of the two alternatives would you choose | Health facility 1  **√** | Health facility 2 |
| --- | --- | --- |

As you can see, in the above example the individual chose ‘Health facility 1’ over ‘Health facility 2’ after comparing the two alternatives. So we can notice that this individual is willing to pay K1700 for a health facility that is within one hour walking distance, has medicines and equipment available and will wait for two hours and will have a superficial examination and will not be treated with respect than getting a free service from a facility which is one and half hours away with thorough examination from respectfully treating staff where there is no medicine and equipment and has waiting time of 5 hours.

Similarly, in the following questions, we will present to you a set of alternatives from which we want you to make a choice. Each alternative is representing a set of attributes which jointly define the health facility from which you may seek care. So we want you to consider these alternatives and tell us which one you would decide to go to with your sick child.

**Choice set 1**

|  | **Health facility 1** | **Health facility 2** |
| --- | --- | --- |
| Distance to health facility | One hour | One and half hours |
| Availability of medicine | Medicine and equipment available | Medicine and equipment not available |
| Waiting time | 3 and half hours | 5 hours |
| Attitude of HW | Treat without respect | Treat with respect |
| Nature of examination | Thorough examination | Thorough examination |
| Cost to you | Free | K 1700 |

| Which of the two alternatives would you choose | Health facility 1 | Health facility 2 |
| --- | --- | --- |

**Choice set 2**

|  | **Health facility 1** | **Health facility 2** |
| --- | --- | --- |
| Distance to health facility | One hour | One and half hours |
| Availability of medicine | Medicine and equipment not available | Medicine and equipment available |
| Waiting time | 2 hours | 5 hours |
| Attitude of HW | Treat with respect | Treat without respect |
| Nature of examination | Superficial examination | Not examined |
| Cost to you | K 1700 | Free |

| Which of the two alternatives would you choose | Health facility 1 | Health facility 2 |
| --- | --- | --- |

**Choice set 3**

|  | **Health facility 1** | **Health facility 2** |
| --- | --- | --- |
| Distance to health facility | Half an hour | One and half hours |
| Availability of medicine | Medicine and equipment not available | Medicine and equipment not available |
| Waiting time | 5 hours | 2 hours |
| Attitude of HW | Treat without respect | Treat with respect |
| Nature of examination | Superficial examination | Through examination |
| Cost to you | K 600 | Free |

| Which of the two alternatives would you choose | Health facility 1 | Health facility 2 |
| --- | --- | --- |

**Choice set 4**

|  | **Health facility 1** | **Health facility 2** |
| --- | --- | --- |
| Distance to health facility | One and half hours | Half an hour |
| Availability of medicine | Medicine and equipment available | Medicine and equipment not available |
| Waiting time | 2 hours | 3 and half hours |
| Attitude of HW | Treat without respect | Treat with respect |
| Nature of examination | Not examined | Not examined |
| Cost to you | K 600 | Free |

| Which of the two alternatives would you choose | Health facility 1 | Health facility 2 |
| --- | --- | --- |

**Choice set 5** *(Hold Out)*

|  | **Health facility 1** | **Health facility 2** |
| --- | --- | --- |
| Distance to health facility | One and half hours | 30 minutes (half hour) |
| Availability of medicine | Medicine and equipment available | Medicine and equipment not available |
| Waiting time | 3 and half hours | 2 hours |
| Attitude of HW | Treat without respect | Treat with respect |
| Nature of examination | Not examined | Thorough examination |
| Cost to you | Free | K 1700 |

| Which of the two alternatives would you choose | Health facility 1 | Health facility 2 |
| --- | --- | --- |

**Choice set 6**

|  | **Health facility 1** | **Health facility 2** |
| --- | --- | --- |
| Distance to health facility | One and half hours | Half an hour |
| Availability of medicine | Medicine and equipment available | Medicine and equipment not available |
| Waiting time | 3 and half hours | 2 hours |
| Attitude of HW | Treat with respect | Treat without respect |
| Nature of examination | Superficial examination | Not examined |
| Cost to you | K 600 | K 1700 |

| Which of the two alternatives would you choose | Health facility 1 | Health facility 2 |
| --- | --- | --- |

**Choice set 7** *(Hold out)*

|  | **Health facility 1** | **Health facility 2** |
| --- | --- | --- |
| Distance to health facility | One and half hours | One hour |
| Availability of medicine | Medicine and equipment available | Medicine and equipment not available |
| Waiting time | 3 and half hours | 5 hours |
| Attitude of HW | Treat with respect | Treat with respect |
| Nature of examination | Superficial examination | Thorough examination |
| Cost to you | Free | K 1700 |

| Which of the two alternatives would you choose | Health facility 1 | Health facility 2 |
| --- | --- | --- |

**Choice set 8**

|  | **Health facility 1** | **Health facility 2** |
| --- | --- | --- |
| Distance to health facility | One hour | Half hours |
| Availability of medicine | Medicine and equipment not available | Medicine and equipment available |
| Waiting time | 3 and half hours | 2 hours |
| Attitude of HW | Treat with respect | Treat with respect |
| Nature of examination | Not examined | Superficial examination |
| Cost to you | Free | K 600 |

| Which of the two alternatives would you choose | Health facility 1 | Health facility 2 |
| --- | --- | --- |

**Choice set 9**

|  | **Health facility 1** | **Health facility 2** |
| --- | --- | --- |
| Distance to health facility | One hour | Half an hour |
| Availability of medicine | Medicine and equipment not available | Medicine and equipment available |
| Waiting time | 5 hours | 3 and half hours |
| Attitude of HW | Treat without respect | Treat without respect |
| Nature of examination | Superficial examination | Through examination |
| Cost to you | Free | K 1700 |

| Which of the two alternatives would you choose | Health facility 1 | Health facility 2 |
| --- | --- | --- |

**Choice set 10**

|  | **Health facility 1** | **Health facility 2** |
| --- | --- | --- |
| Distance to health facility | Half an hour | One and half hours |
| Availability of medicine | Medicine and equipment available | Medicine and equipment not available |
| Waiting time | 5 hours | 3 and half hours |
| Attitude of HW | Treat with respect | Treat without respect |
| Nature of examination | Through examination | Superficial examination |
| Cost to you | K 600 | K 1700 |

| Which of the two alternatives would you choose | Health facility 1 | Health facility 2 |
| --- | --- | --- |

**Choice set 11**

|  | **Health facility 1** | **Health facility 2** |
| --- | --- | --- |
| Distance to health facility | One hour | One hour |
| Availability of medicine | Medicine and equipment not available | Medicine and equipment available |
| Waiting time | 2 hours | 5 hours |
| Attitude of HW | Treat without respect | Treat with respect |
| Nature of examination | Through examination | Not examined |
| Cost to you | K 600 | K 1700 |

| Which of the two alternatives would you choose | Health facility 1 | Health facility 2 |
| --- | --- | --- |

**12** How easy or difficult did you find the section on choice sets

1. Very Easy
2. Easy
3. Difficult
4. Very Difficult
5. Extremely Difficult

**Section III Socio-demographic characteristics**

Please answer the following questions about yourself. Although some of these questions are personal, the information you give is confidential.

| 1. How old is the respondent | ___________ | |
| --- | --- | --- |
| 1. Gender of respondent? | Male  Female | |
| 1. Marital status | Never married |  |
|  | Married |  |
|  | Divorced/separated |  |
|  | Widow widowed |  |
| 1. How many children? | 1. One child 2. Two children 3. Three children 4. Four children 5. Five or more children | |
| 1. What is the level of respondent’s education? | 1. No education 2. Primary education 3. Secondary education 4. Tertiary education | |
| 1. What is the estimated monthly household income? | ______________________________ | |
|  |  | |
| 1. What is your occupation? 2. Sex of the child | 1. Government employee at professional level 2. Government employee at junior level 3. Self employed doing small scale business 4. Casual work / labour 5. Student 6. Private sector at professional level 7. Private sector at junior level 8. Farming 9. No activity 10. Male 11. Female | |
